# 2283 Utilization of ClinicalTrials.gov registry to demonstrate the extent of dissemination bias in anesthesiology

**DOI:** 10.1017/cts.2018.310

**Published:** 2018-11-21

**Authors:** Singh Nair, Davis Johns, Ellise Delphin, Jonathan Leff

**Affiliations:** Icahn School of Medicine at Mount Sinai

## Abstract

OBJECTIVES/SPECIFIC AIMS: The purpose of this study is to evaluate the extent of publication bias in anesthesia and to evaluate the characteristics of studies that are registered and unpublished. METHODS/STUDY POPULATION: We used the advanced search option and the key word “anesthesia” to identify anesthesia related studies in the ClinicalTrials.gov registry. For the purpose of this analysis we have randomly selected 50% of the anesthesia related studies from the year 2008–2013. We have collected information pertaining to drug/device study, origin, type, design, subspecialty, enrollment target, anesthesia type, and adult/ pediatric, sponsored/ investigator initiated, population studied and start and end date. Studies with an ongoing, terminated, or unknown status were excluded from the analysis. For results, we initially searched the results section associated with each study; also we searched for any publication link at the study result area of the registry. For studies with no results and publication links we searched on PubMed, Google Scholar, and Embase by trial registration number, study title, and investigators name for matching manuscripts. In addition, we also analyzed the proportion of studies with positive and negative conclusions. We used descriptive and univariate statistics to report the results. RESULTS/ANTICIPATED RESULTS: Overall, 5448 studies were identified within the queried timeframe. We have included 2649 studies in our final analysis and detailed analysis were performed for 1778 studies with the status “completed.” The mean, standard deviation of subjects enrolled in completed trials was 392.47±6378. Only 162 (9.9%) studies registered were in the pediatric population, and 1616 (90.9%) were in the adult population. Finally, of the reviewed studies, 1486 (83.6%) were investigator-initiated, 207 (11.6%) were sponsored, and 85 (4.8%) were registered as collaborated studies. Among the completed studies only 296 (16.6%) studies posted results to the result section of the registry. Additionally, a link associated with a publication was posted in only 393 (22.1%) of the studies. The proportion of studies with posted results were 208 (14%), 61 (29%) and 27 (31.8%) in investigator-initiated, sponsored, and collaborated studies *p*<0.001 respectively. In the 1778 studies we reviewed, 954 (53.7%) studies were associated with one publication. In the published studies, 721 (75.6%) studies reported a positive conclusion for their publication. DISCUSSION/SIGNIFICANCE OF IMPACT: Only, 53.7% of anesthesia related studies with a “complete” status in ClinicalTrials.gov were published. Furthermore, investigators fail to fulfill the requirement of making the results available in the results section of the registry. Lack of availability of published literature and the nonavailability of the results from these studies contributes to publication bias and also failure to honor the ethical responsibility of the investigator to share the results of the study with subjects and with the medical community around the world.

